# Lipid a remodeling modulates outer membrane vesicle biogenesis by *Porphyromonas gingivalis*

**DOI:** 10.1128/jb.00336-24

**Published:** 2024-12-11

**Authors:** Sarah R. Alaei, Alisa J. King, Karim Banani, Angel Reddy, Joshua Ortiz, Alexa L. Knight, Jessica Haldeman, Thet Hnin Su, Hana Park, Stephen R. Coats, Sumita Jain

**Affiliations:** 1 Division of Science and Mathematics, School of Interdisciplinary Arts and Sciences, University of Washington 7284 , Tacoma, Washington, USA; 2 Department of Periodontics, School of Dentistry, University of Washington 7284 , Seattle, Washington, USA; University of Notre Dame, Notre Dame, Indiana, USA

**Keywords:** outer membrane vesicles, lipid A, *Porphyromonas gingivalis*

## Abstract

Outer membrane vesicles (OMVs) are small membrane enclosed sacs released from bacteria which serve as carriers of biomolecules that shape interactions with the surrounding environment. The periodontal pathogen, *Porphyromonas gingivalis*, is a prolific OMV producer. Here, we investigated how the structure of lipid A, a core outer membrane molecule, influences *P. gingivalis* OMV production, OMV-dependent TLR4 activation, and biofilm formation. We examined mutant strains of *P. gingivalis* 33277 deficient for enzymes that alter lipid A phosphorylation and acylation status. The lipid A C4′-phosphatase (*lpxF*)-deficient strain and strains bearing inactivating point mutations in the LpxF active site displayed markedly reduced OMV production relative to WT. In contrast, strains deficient for either the lipid A C1-phosphatase (*lpxE*) or the lipid A deacylase (PGN_1123; *lpxZ*) genes did not display alterations in OMV abundance compared to WT. These data indicate that lipid A C4′-phosphate removal is required for typical OMV formation. In addition, OMVs produced by *ΔlpxF* and *ΔlpxZ* strains, possessing only penta-acylated lipid A, stimulated robust TLR4 activation, whereas OMVs obtained from WT and *ΔlpxE* strains, containing predominantly tetra-acylated lipid A, did not. Hence, lipid A remodeling modulates the capacity of OMVs to engage host TLR4-dependent immunity. Finally, we demonstrate an inverse relationship between OMV abundance and biofilm density, with the *∆lpxF* mutants forming denser biofilms than either WT, *ΔlpxE*, or *ΔlpxZ* strains. Therefore, OMVs may also contribute to pathogenesis by regulating biofilm formation and dispersal.

**IMPORTANCE:**

*Porphyromonas gingivalis* is a bacterium strongly associated with periodontitis. *P. gingivalis* exports lipids, proteins, and other biomolecules that contribute to the bacterium’s ability to persist in inflammatory conditions encountered during disease. These biomolecules are exported through several mechanisms, including via outer membrane vesicles (OMVs). Despite their ubiquity, the mechanisms that drive outer membrane vesicle production vary among bacteria and are not fully understood. In this study, we report that C4′ dephosphorylation of lipid A, a major outer membrane molecule, is required for robust outer membrane vesicle production and biological function in *P. gingivalis*. This finding adds to the growing body of evidence that lipid A structure is an important factor in outer membrane vesicle biogenesis in diverse bacterial species.

## INTRODUCTION

*Porphyromonas gingivalis* is a Gram-negative bacterium that is a periodontal pathogen capable of precipitating chronic inflammation. Animal models of *P. gingivalis* infection have highlighted its role in the establishment of microbial dysbiosis and an increase in the overall bacterial load in periodontal tissues, leading to inflammation (1–4), which are characteristic features of periodontitis. As a successful chronic pathogen, *P. gingivalis* utilizes a diverse array of biomolecular tools to manipulate its surroundings in ways that promote its survival (5–7). The abundant outer membrane vesicles (OMVs) produced by *P. gingivalis* have been hypothesized to serve as an important delivery system for enzymes, RNA, and other biomolecules involved in manipulation of the periodontal microenvironment and systemic immune dysregulation (8–17). Given their small size, OMVs can disseminate far from their origin and impart biological functions such as manipulation of host cell responses (18–20), facilitation of communication between bacteria (21), and modulation of biofilm dynamics (22, 23). Thus, OMVs are expected to make significant contributions to the pathogenesis of *P. gingivalis* in periodontal pockets.

**Fig 1:**
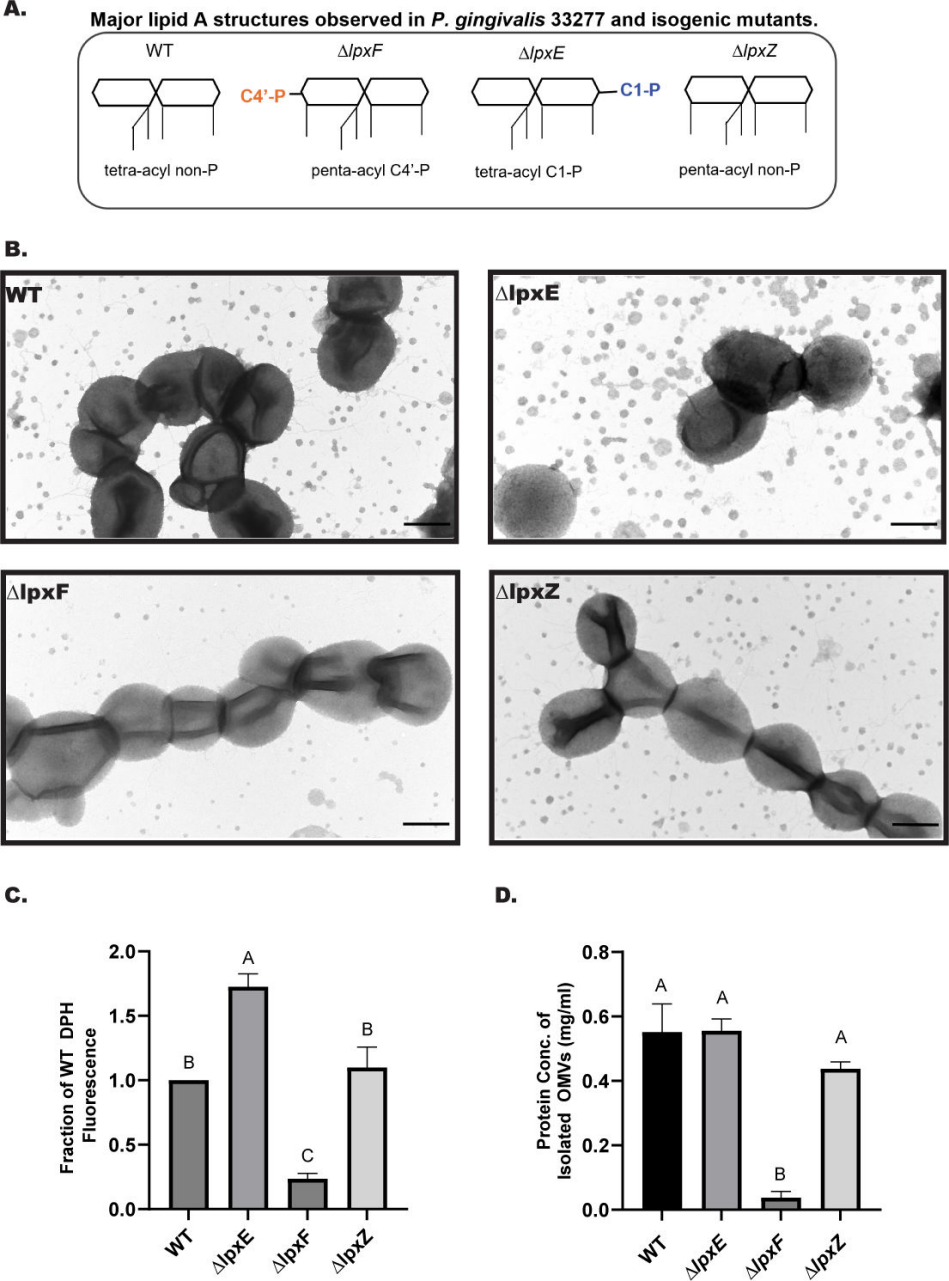
Lipid A C4′ phosphatase (*lpxF*) gene deletion impairs OMV biogenesis. (**A**) Major lipid A structure in *P. gingivalis* wild-type and lipid A modification mutants showing the position of phosphate groups (C1 or C4′) and number of acyl chains. (**B**) TEM images of whole broth cultures (scale bar = 500 nm), (**C**) quantification of relative lipid content in isolated OMVs by DPH assay (*n* = 5 independent OMV isolations), and (**D**) quantification of protein content in isolated OMVs by BCA assay (*n* = 3 independent OMV isolations) were used to compare abundances of OMVs produced by WT 33277 and its lipid A modifying enzyme mutants. Error bars denote the standard error of the mean (SEM), and different letters above bars indicate statistically significant differences between strains (*P* < 0.05) determined by one-way ANOVA and *post hoc* Tukey test.

The membrane that encloses OMVs is derived from the outer membrane (OM) and, thus, enriched with lipopolysaccharide (LPS), which comprises the outer layer of the OM. Lipid A is the hydrophobic anchor of LPS, which is heterogenous in structure in *P. gingivalis* due to enzymatic modifications that occur after LPS biosynthesis (24–27). Lipid A of *E. coli*, for example, has one predominant structure comprised by a di-glucosamine residue with six acyl chains and two phosphate groups attached (28, 29). Lipid A of *P. gingivalis*, on the other hand, can be penta or tetra-acylated, and bis-, mono- or non-phosphorylated (26, 27). The molecule first made, penta-acylated bis-phosphorylated, is subject to modification by three lipid A modifying enzymes leading to the formation of a final tetra-acylated non-phosphorylated structure. Previous studies in our lab have led to the identification of three lipid A modifying genes, namely, the lipid A C1-phosphatase *lpxE* (encoded by PG1773 (26), lipid A C4′-phosphatase *lpxF* (encoded by PG1587 (26, 30, 31), and the lipid A deacylase, which we are naming *lpxZ* [encoded by PGN_1123 (32)]. Deletion mutations in these genes led to the generation of *P. gingivalis* mutants with defined lipid A structure (Fig. 1A).

The impact of *P. gingivalis* LPS, specifically lipid A structures, on the production of OMVs has not been comprehensively studied. Lipid A structure potentially plays a significant role by altering membrane fluidity, charge, associated protein content, and dynamics. OMVs in *P. gingivalis* have been shown to contain primarily under-acylated lipid A suggesting hypo-acylated lipid A facilitates OMV formation (33). Consistent with this hypothesis, Elhanawy et al. demonstrated that OMV formation increased in an *E. coli* mutant over-expressing the *Salmonella* lipid A deacylase gene, which corresponded with an increase in penta- vs hexa-acylated lipid A (34). The suggestion that under-acylated lipid A promotes OMV formation was further validated by the Curtis group by finding ~50% attenuation in OMV production by a *P. gingivalis* mutant, ∆*lptO*, harboring lipid A with more acyl chains than WT, penta- instead of tetra-acylated (35). Taken together, these findings suggest that OMV biogenesis is dependent upon specific lipid A structures.

Here, we investigate the impact of loss of function mutations in the lipid A remodeling enzymes LpxE, LpxF, and LpxZ on OMV biogenesis in *P. gingivalis* 33277. We demonstrate that OMV production by the lipid A deacylase mutant, Δ*lpxZ,* which has penta-acyl lipid A is not impaired for OMV formation. In contrast, the lipid A C4′-phosphatase mutant, ∆*lpxF*, which like Δ*lpxZ* also has lipid A locked in a penta-acylated form but is C4′-phosphorylated, is heavily impaired for OMV formation, indicating removal of C4′-phosphate is required for optimal OMV formation. The lipid A C1-phosphatase mutant ∆*lpxE*, on the other hand, makes the same or more OMVs than wild-type demonstrating opposite effects by the two phosphatase mutants. From a functional standpoint, we demonstrate that OMVs made by ∆*lpxF* stimulate the innate immune receptor TLR4 more than wild-type OMVs similar to TLR4 activation by their corresponding whole bacteria. We also demonstrate that the reduction of OMV production by ∆*lpxF* correlates with an increase in biofilm density, suggesting that *P. gingivalis* OMVs are an important modulator of biofilm dynamics, which could contribute to bacterial survival and induction of dysbiosis in the host.

## RESULTS

### Deletion of *lpxF*, the lipid A C4′-phosphatase encoding gene, impairs OMV production in *P. gingivalis* 33277

To examine the effects of lipid A structure on OMV production by *P. gingivalis,* we compared relative abundances of OMVs released from WT 33277 and isogenic mutants with deletions of genes encoding lipid A modifying enzymes, which display distinct predominant lipid A structures (Fig. 1A). We have previously shown that lipid A in WT 33277 is largely tetra-acylated, non-phosphorylated (26, 27). In contrast, lipid A of the deacylase mutant, *ΔlpxZ*, is primarily penta-acylated, non-phosphorylated (32), lipid A of the C1-phosphatase mutant, *ΔlpxE*, is mostly tetra-acylated, C1-phosphorylated and lipid A of the C4′-phosphatase mutant, *ΔlpxF*, is penta-acylated, C4′ phosphorylated (26, 30) (Fig. 1A). We hypothesized that the penta-acylated lipid A mutants lacking lipid A deacylase or C4’-phosphatase genes would display differing levels of OMV production compared to their parental WT strain.

Transmission electron microscopy (TEM) images, shown in Fig. 1B, revealed a striking reduction in OMV production by the *∆lpxF* mutant compared to WT, *ΔlpxE,* and *ΔlpxZ* mutants. The *∆lpxF* C4′-phosphatase mutant possesses lipid A that is C4′-mono-phosphorylated as expected but also penta-acylated (26, 30, 36). Deletion of the lipid A deacylase gene (*lpxZ*) also results in penta-acylated lipid A (32). However, this mutation does not significantly change the abundance of OMVs produced, indicating that deacylation is not a prerequisite for OMV production in *P. gingivalis*. In other words, penta-acylated mutants can make WT levels of OMVs, but it is the presence of C4′-phosphate that restricts OMV formation. The phosphatase mutant lacking the C1-phosphatase gene, *lpxE*, produced OMVs similar in number or higher than WT (Fig. 1B). To confirm the relative levels of OMV production by each strain that we had observed by TEM, OMVs were isolated from cell-free culture supernatants by ultra-centrifugation. Lipid and protein content of the isolated OMVs were quantified using a lipophilic probe (37) and BCA assay, respectively. Both lipid (Fig. 1C) and protein content (Fig. 1D) were significantly reduced in the OMV pellets isolated from the ∆*lpxF* mutant relative to WT, *∆lpxE*, and *ΔlpxZ,* consistent with the reduced abundance of OMVs observed by TEM.

To confirm that there is a general, and not strain specific, requirement for LpxF activity to generate robust OMV production in *P. gingivalis,* we constructed isogenic *ΔlpxF* and *ΔlpxE* mutants in the *P. gingivalis* strains 381 (38) and W83. Whole-genome sequence and RNAseq analyses have revealed that *P. gingivalis* strain 381 is very similar to 33277 (39, 40). However, while both strains are non-capsulated, they exhibit differences in gingipain activity and TLR2 stimulation (41). OMV production by strain 381 WT and isogenic single mutants *ΔlpxE* and *ΔlpxF* by TEM (Fig. S1) revealed that *ΔlpxF* displayed reduced OMV production compared to WT. Strain W83 is a capsulated strain with substantial genetic and phenotypic differences compared to 33277 (42, 43). However, comparison of OMV production by WT W83 and its isogenic single mutants *ΔlpxE* and *ΔlpxF* revealed the same trend of reduced OMVs by ∆*lpxF* C4′-phosphatase mutant, suggesting a conserved role for LpxF in OMV production among *P. gingivalis* strains (Fig. S1).

### Point mutations in the active site of C4′-phosphatase also impair OMV production

**Fig 2:**
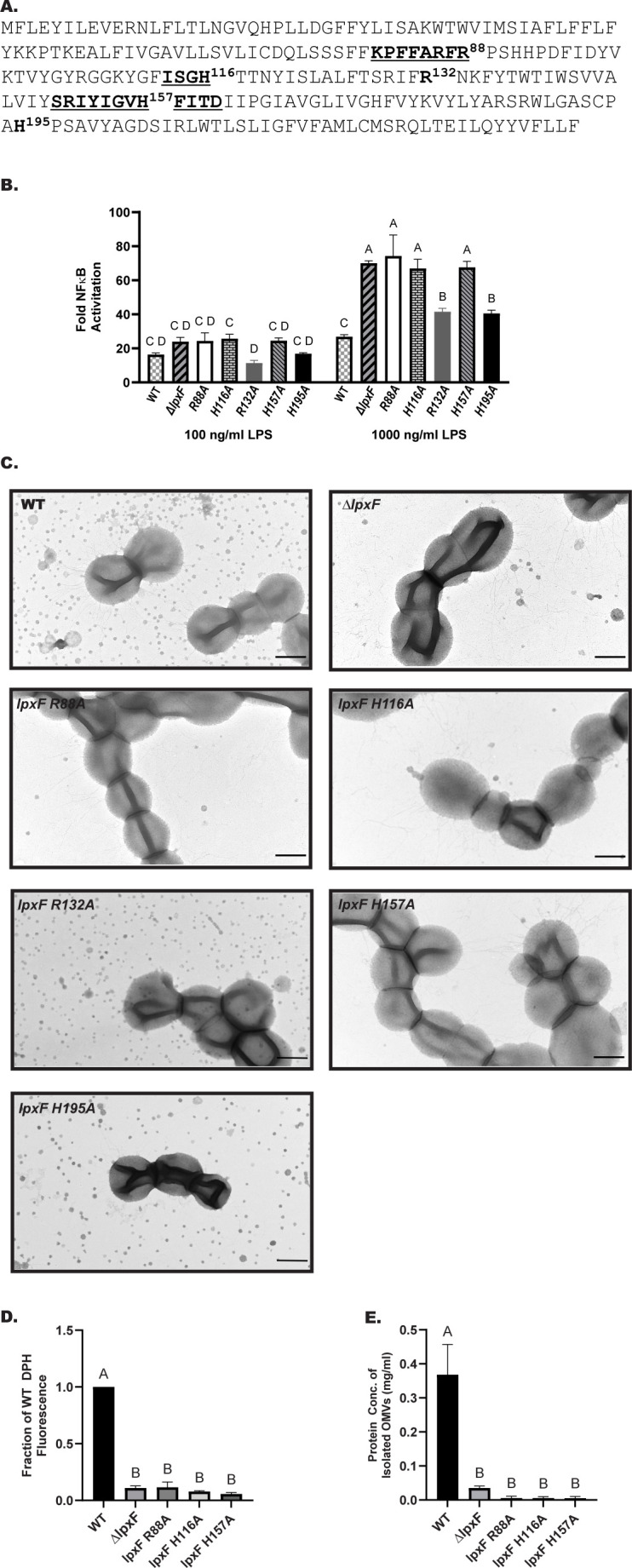
Point mutants with a predicted loss of function in the *lpxF* gene display impaired OMV production. (**A**) Protein sequence of LpxF in *P. gingivalis* 33277, with PAPII domain underlined and in bold. Residues targeted for mutagenesis are shown in bold along with the residue number. (**B**) Deletion and point mutants in *lpxF* stimulate TLR4. HEK293 cells expressing TLR4/MD-2 were stimulated with LPS isolated from the depicted mutants. Fold NF-kB stimulation of infected cells relative to the unstimulated control is plotted on the *y-*axis. The results are means ± standard deviation (SD) of triplicate samples from one of two independent experiments. Different letters above bars indicate statistically significant differences between treatments (*P* < 0.05) determined by two-way ANOVA and post hoc Tukey test. (**C**) TEM images of whole broth cultures (scale bar = 500 nm), (**D**) quantification of lipid content in OMVs by DPH assay (*n* = 3 independent OMV isolations), and (**E**) quantification of protein content in isolated OMVs by BCA assay (*n* = 3 independent OMV isolations). The results are means +/− SEM, and different letters above bars indicate statistically significant differences between strains (*P* < 0.05) determined by one-way ANOVA and *post hoc* Tukey test.

The *lpxF* gene, PGN_0524 in *P. gingivalis 33277*, is annotated to encode a conserved PAP2 domain for Type 2 phosphatidic acid phosphatase activity similar to that seen in the *lpxF* gene in *Francisella novicida* (44, 45). To determine if this represents the active site for C4′-phosphatase activity in *P. gingivalis* LpxF, we constructed individual site-directed point mutations in the PAP2 domain, changing three amino acids (bold and underlined, Fig. 2A), as well as two residues outside the PAP2 domain (bold without underline, Fig. 2A), to alanine residues. Lipid A structural and functional analyses revealed that each amino acid substitution within the PAP2 domain, namely R88A, H116A, and H157A, displayed a lipid A phenotype characteristic of the ∆*lpxF* mutant. Lipid A isolated from these mutants is penta-acylated C4′-phosphorylated, as revealed by MALDI-TOF mass spectrometry (Fig. S2). The shift from primarily tetra-acylated unphosphorylated lipid A in WT to penta-acylated C4′ phosphorylated lipid A in the *lpxF* deletion and R88A, H116A, and R157A point mutants led to a concomitant increase in stimulation of the innate immune receptor TLR4 as shown by a HEK TLR4 assay (Fig. 2B). On the other hand, single amino acid substitutions located outside the PAP2 domain (R132A, H195A) exhibited lipid A structure (Fig. S2) and TLR4 activation (Fig. 2B) similar to WT. We hence confirmed that the PAP2 domain represents the active site of the C4′-phosphatase enzyme, and mutation of a single amino acid within this motif changes lipid A from a tetra-acylated non-phosphorylated TLR4-evasive structure to a penta-acylated C4′-phosphorylated TLR4-stimulating structure similar to that seen in the ∆*lpxF* mutant. The three active site mutants were sensitive to polymyxin B as well (50 µg/mL, data not shown) similar to the ∆*lpxF* deletion mutant (26, 30).

TEM of *lpxF* active site point mutants revealed a substantial reduction in OMV production, similar to the ∆*lpxF* deletion mutant, whereas mutations outside of the PAP2 domain did not impair OMV production (Fig. 2C). The OMV production phenotype of the *lpxF* active site mutants observed by TEM was validated by evaluation of protein and lipid content of OMVs isolated from culture supernatants, which were significantly reduced in all three *lpxF* active-site mutants compared to WT (Fig. 2D and E). These results confirmed that the phosphatase activity of LpxF is required for OMV formation as opposed to a potentially different function mediated by LpxF.

### OMVs from penta-acylated lipid A mutants display increased TLR4 stimulation compared to WT

**Fig 3:**
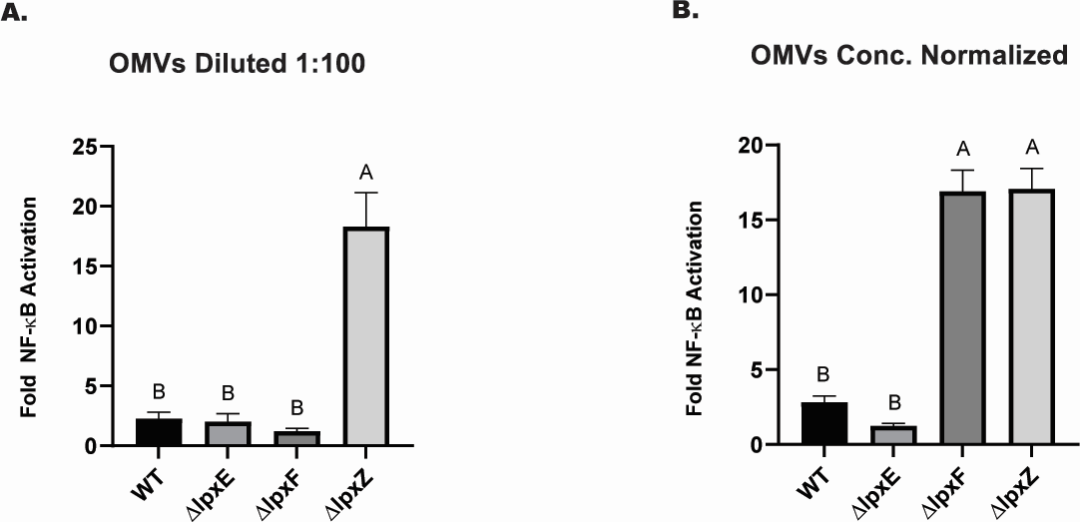
OMVs from ∆*lpxZ* and ∆*lpxF* penta-acylated lipid A mutants stimulate TLR4. HEK293 cells expressing TLR4/MD-2 were exposed to (**A**) 1:100 dilution of OMVs isolated from wild-type 33277 and isogenic lipid A modification mutants, and (**B**) OMVs that were normalized by quantity to lipid content of wild-type OMVs as measured by DPH assay. Fold NF-kB stimulation of infected cells relative to the unstimulated control is plotted on the *y-*axis. The results are means ± SD of triplicate samples from one of two independent experiments and different letters above bars indicate statistically significant differences between treatments (*P* < 0.05) determined by one-way ANOVA and *post hoc* Tukey test.

Given the potential role of OMVs as modulators of host-pathogen interactions (8, 9), we compared TLR4 stimulation by OMVs from WT, *∆lpxF, ΔlpxE,* and *ΔlpxZ* mutants using a HEK293 TLR4 assay (32, 41). Following transfection of the non-immune HEK293 cells with plasmids encoding TLR4, MD-2, and NF-κB luciferase reporter genes, the cells were treated with OMVs. Addition of a 1:100 dilution of OMVs isolated from bacterial culture supernatants grown to equal cell densities revealed increased stimulation only by the *ΔlpxZ* mutant, relative to WT (Fig. 3A). The *ΔlpxZ* mutant OMVs contain LPS that is locked in the penta-acylated form due to the absence of lipid A deacylase, and our previous work has shown that penta-acylated lipid A stimulates TLR4 in contrast to tetra-acylated lipid A which is TLR4 evasive (26, 27, 32). Next, OMV preparations were normalized so that mutant OMVs were equivalent in amount to WT OMVs by the measure of lipid content from the DPH assay. After normalization, which led to the addition of ~4-fold more *∆lpxF* OMVs, an increase in TLR4 stimulation by *∆lpxF* was detected as well when compared to OMVs from WT and *∆lpxE* mutants (Fig. 3B). Additionally, OMVs isolated from strains with single amino acid substitutions within the LpxF PAP2 domain elicited increased TLR4 stimulation compared to WT or strains with amino acid substitution outside of the LpxF PAP2 domain (Fig. S3). Hence, these results indicate that OMVs made by mutants with penta-acylated lipid A are more pro-inflammatory than OMVs from strains with tetra-acylated lipid A, similar to the whole bacterial strains from which they are derived.

Since *P. gingivalis* is also known to stimulate TLR2, we examined human TLR2 + TLR1 activation in the presence of the CD14 co-receptor by *P. gingivalis* OMVs using a HEK293-based assay (46–48). Following transfection of HEK293 cells with plasmids encoding TLR2, TLR1, CD14, and NF-κB luciferase reporter genes, the cells were exposed to OMVs isolated from WT *P. gingivalis 33277* and isogenic *∆lpxF, ΔlpxE,* and *ΔlpxZ* mutants. Fig. S4 demonstrates that OMVs activate TLR2 + TLR1 robustly, and to a much greater extent than they activate TLR4, indicating OMVs are loaded with agonists of the TLR2 + TLR1 heterodimer. We did not observe a significant difference in TLR2 + TLR1 activity when comparing HEK293 cells treated with OMVs derived from WT vs the isogenic *∆lpxF*, *ΔlpxE*, and *ΔlpxZ* mutants.

### OMVs regulate biofilm density

OMVs have been observed within the extracellular matrices of biofilms (46) and contain lipids, polysaccharides, DNA, and proteins that are commonly found in the extracellular matrices of biofilms. Comparison of OMV and extracellular matrix proteomes of biofilms suggest that OMVs are a major source of extracellular matrix proteins such as adhesins and metabolic enzymes in biofilms (22, 49–51). The delivery of biofilm matrix components via OMVs potentially underlies the positive correlation between OMVs and biofilm formation observed in species such as *Helicobacter pylori* (52), *Aeromonas veronii* (22), and *Pseudomonas putida* (53). However, OMVs can also promote biofilm dispersal, as observed in *Pseudomonas* aeruginosa, through the delivery of enzymes that degrade extracellular matrix components such as proteins, lipids, and DNA (23).

Based on these previously identified roles for OMVs in biofilm formation and dispersal, we hypothesized that *P. gingivalis* strains producing different quantities of OMVs would produce biofilms with differing densities or architectures. If *P. gingivalis* biofilm formation is positively regulated with OMV production, then we would expect to observe denser biofilms formed by strains that produce more abundant OMVs. Alternatively, if OMVs promote biofilm dispersal and/or inhibit cell adhesion, then we would expect denser biofilms formed by the strains producing fewer OMVs. To test this hypothesis, we allowed *P. gingivalis* cells to form biofilms for 48 h on glass coverslips and then quantified biofilm density by determining the integrated pixel intensity of confocal image stacks for each strain or treatment group.

**Fig 4:**
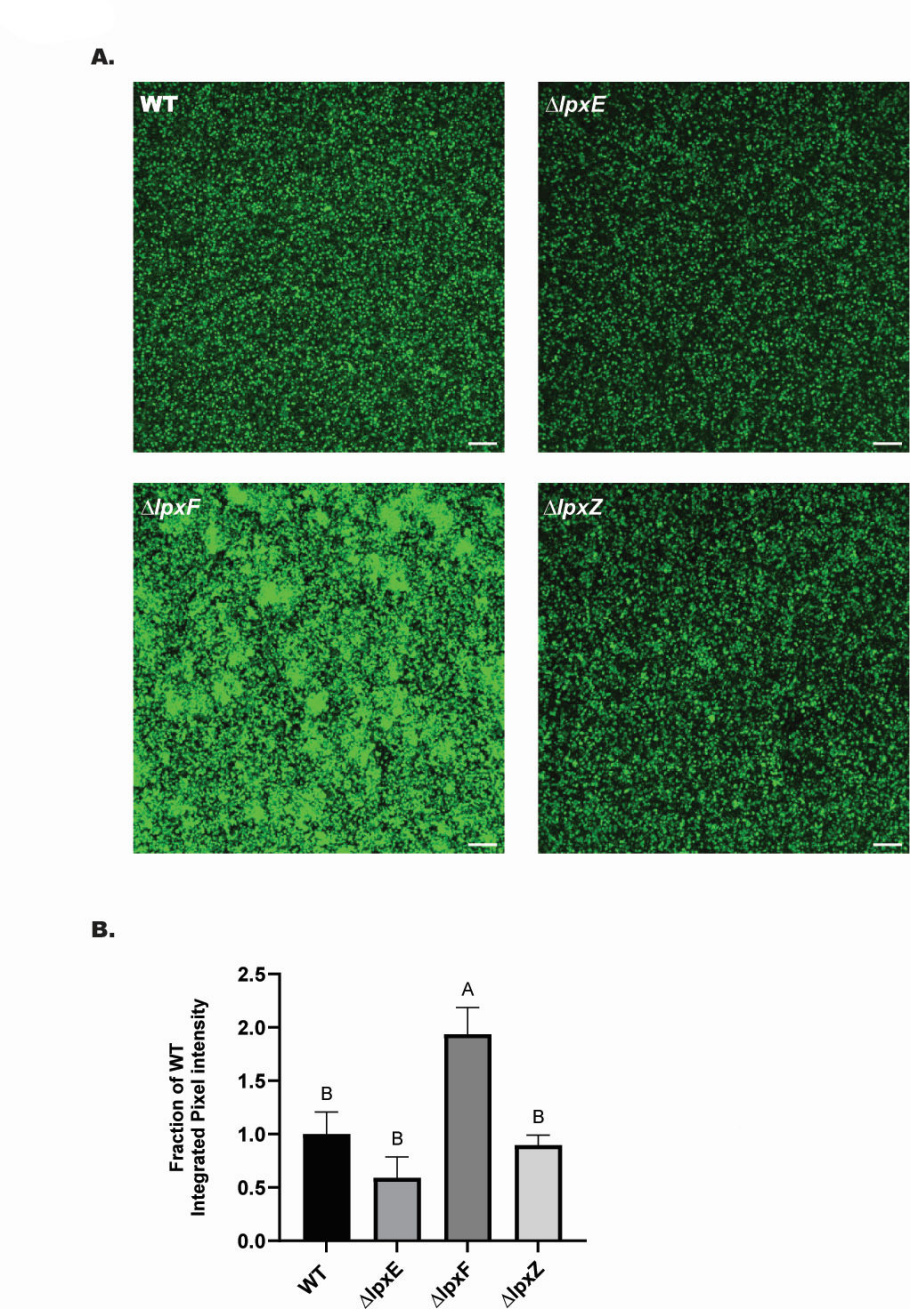
OMV abundance is inversely related to biofilm density. Biofilms were formed by CFSE labeled *P. gingivalis* on glass coverslips for 48 h. (**A**) Representative Z-projections of confocal image stacks (scale bar = 10 µm) are shown. (**B**) Integrated green pixel intensities were determined for each Z-stack and normalized to WT for each experiment. Bars represent means ± SEM for four images per coverslip, with six coverslips analyzed per strain. Different letters above bars indicate statistically significant differences between strains (*P* < 0.05) determined by one-way ANOVA and *post hoc* Tukey test.

**Fig 5:**
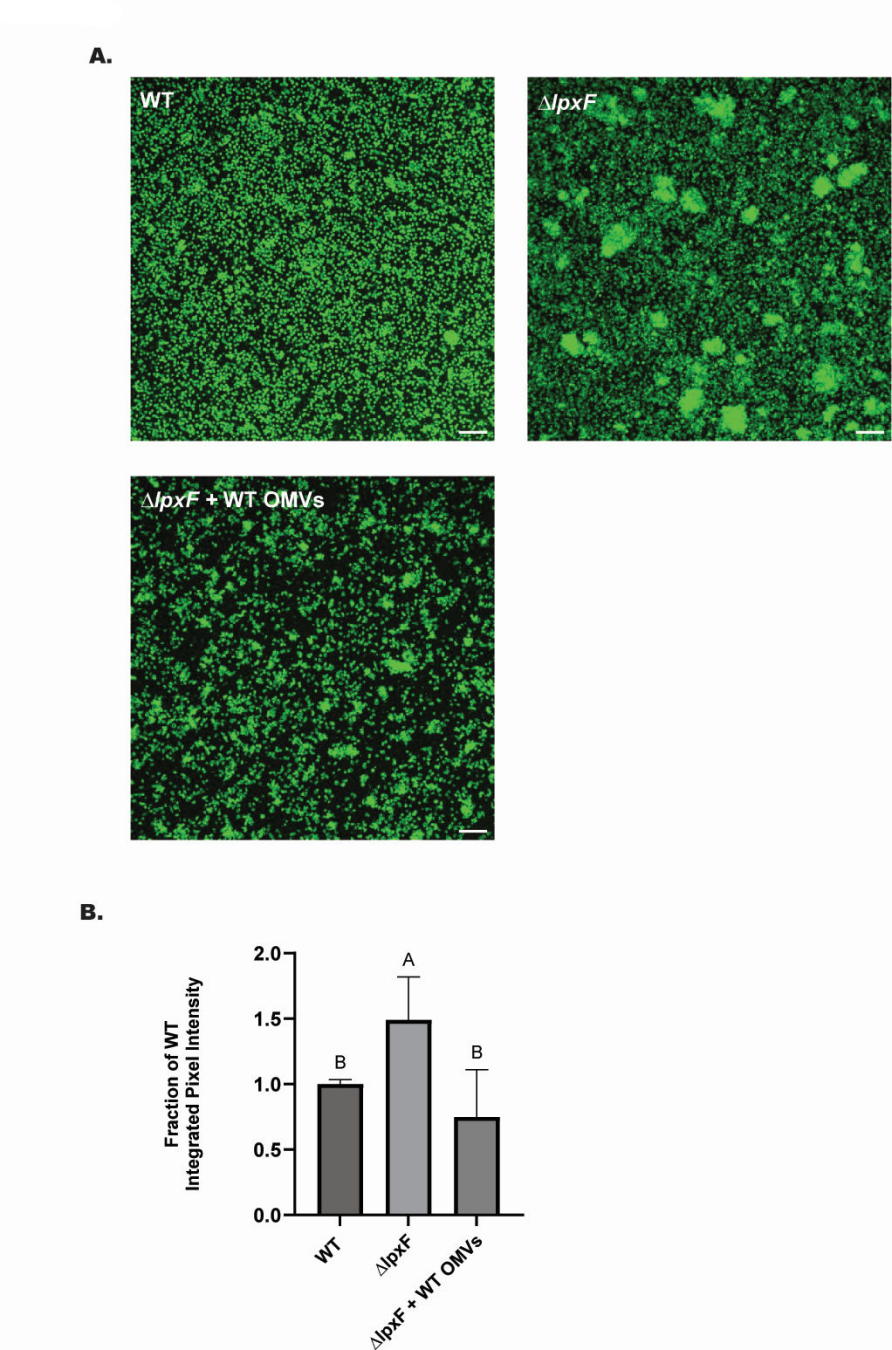
Addition of WT OMVs reduces density of *∆lpxF* biofilms. CFSE-labeled *P. gingivalis* cells were used to form biofilms on glass coverslips for 48 h. WT OMVs isolated from stationary phase cultures were added to *∆lpxF* prior to biofilm formation where indicated. (**A**) Representative Z-projections of confocal image stacks with 10 µm scale bars. (**B**) Integrated green pixel intensities were determined for each Z-stack and normalized to WT for each experiment. Bars represent means ± SEMs of four images per coverslip with six coverslips analyzed per strain. Different letters above bars indicate statistically significant differences between strains (*P* < 0.05) determined by one-way ANOVA and *post hoc* Tukey test.

Our results, as seen in Fig. 4 and 5, indicate that OMV abundance is negatively correlated with biofilm density in *P. gingivalis*. The WT and *∆lpxZ* strains, which produced similar amounts of OMVs (Fig. 1B through D), produced similarly dense biofilms (Fig. 4). The *∆lpxE* mutant displayed a trend toward lower biofilm density compared to WT (Fig. 4), which corresponded to slightly increased OMV production (Fig. 1B through D). However, *∆lpxF*, which displays severely impaired OMV production compared to WT (Fig. 1B through D), formed a much denser biofilm (Fig. 4). Supplementation of *∆lpxF* cultures with WT OMVs during biofilm formation reduced *∆lpxF* biofilm density (Fig. 5). Taken together, these results demonstrate that the impact of *P. gingivalis* lipid A structure on OMV counts plays a significant role in biofilm density with strains producing lower number of OMVs exhibiting higher biofilm density.

## DISCUSSION

In this study, we investigated the impact of lipid A structural features on OMV formation in *P. gingivalis*. As an integral part of LPS, lipid A is the principal molecule forming the outer layer of the outer membrane, and hence of outer membrane vesicles. It is known to be the bio-active moiety of LPS recognized by the pattern-recognition immune receptor, TLR4. Our previous work on this feature of lipid A, recognition by TLR4, led to elucidation of *P. gingivalis* lipid A structure, comprised by a di-glucosamine residue to which are attached 4–5 acyl chains and 0–2 phosphate groups (25, 26, 54). We identified the two lipid A phosphatases (26) and the lipid A deacylase enzyme (32), of which the C4′-phosphatase and deacylase are required for the formation of tetra-acylated lipid A structures, leading to evasion of the TLR4 response. The C4′-phosphatase is also required for resistance to the cationic antimicrobial peptide polymyxin B indicating that the presence of the negatively charged C4′-phosphate molecule is necessary for polymyxin B-dependent formation of pores in the OM leading to loss in viability (30).

The C1- and C4′-phosphatase mutants display distinctly contrasting phenotypes pertaining to TLR4 activation—antagonistic vs agonistic, respectively (26, 38), and to growth in the presence of polymyxin B—resistance vs sensitivity (30). We now demonstrate that the dichotomy extends to vesiculation since the C4′-phosphatase mutant, ∆*lpxF,* displays a significant reduction in the abundance of OMVs produced and the C1-phosphatase mutant, ∆*lpxE,* displays a trend toward higher vesiculation than wild-type. Differential packaging dynamics of fatty acyl chains, decreased binding to the underlying peptidoglycan, increased binding to cationic molecules or other factors that remain unidentified, could be contributing to the reduced vesiculation observed in the ∆*lpxF* mutant and remain to be investigated.

Previous investigations in *P. gingivalis* (35) and *Salmonella* (34) had suggested that mutations resulting in increased lipid A acyl chain number relative to wild type reduced OMV production, or vice versa. However, we now show that the lipid A deacylase mutant, ∆*lpxZ*, which harbors only penta-acylated lipid A, is not compromised for OMV production relative to wild type, which has mostly tetra-acylated lipid A in its OM. It is important to note that the lipid A in the C4′-phosphatase mutant, *∆lpxF*, is locked in a penta-acylated form as well, indicating the C4′-phosphate needs to be removed for deacylation of lipid A to occur. The only difference between the lipid A found in ∆*lpxF* and ∆*lpxZ* mutants is that the former is phosphorylated on C4′, while the latter has a mix of mono-, bis-, and non-phosphorylated lipid A structures. Taken together, these findings indicate that vesiculation in *P. gingivalis* strain 33277 is impaired by the presence of phosphate exclusively in the C4′-position and not by the presence of penta-acylated lipid A.

Our initial observations with Pg strain 33277 mutants were validated with Pg 381 since its ∆*lpxF* and ∆*lpxE* mutant strains displayed similar vesiculation phenotypes as the corresponding mutants in 33277, low and high, respectively. Both Pg 33277 and 381 are not capsulated, and while they have a very similar whole-genome sequence (39, 40, 42), they are significantly different in terms of virulence (41, 55). Our preliminary analysis of the ∆*lpxE* and ∆*lpxF* mutants in the capsulated strain Pg W83 revealed the same trend in that the ∆*lpxF* mutant is impaired for OMV formation while ∆*lpxE* is not. However, it is worth noting that a study in the capsulated strain Pg W50 reported that a mutant with impaired lipid A C1- phosphatase activity displayed a significant reduction in OMV production compared to WT (35). This mutant was generated by deleting the *lptO* gene, which does not encode a lipid A modifying enzyme. It remains to be determined whether the impaired OMV production in Pg W50 ∆lptO results from a strain-specific impact of lipid A C1-phosphatase activity or whether there are additional substrates or interacting factors of LptO that regulate OMV production in Pg W50.

In addition to implicating C4′-lipid A phosphatase activity as a regulator of OMV production, we also provided support for a conserved PAP2 active site in LpxF in *P. gingivalis*. It is possible that point mutations constructed in the PAP2 domain (*lpxF* R88A, *lpxF H116A*, *lpxF H157A*) are expressing unstable forms of LpxF compared to WT, thus becoming the equivalent of the *∆lpxF* mutant. However, we have several reasons for assuming that the LpxF PAP2 domain mutants are expressing a stable, but enzymatically inactive forms of LpxF. We were not able to use Western blotting or some other protein detection method to confirm that the five mutant proteins expressed by the mutants in our study were stable and present at levels equivalent to WT LpxF, but a previous study in *Rhizobium leguminosarum* revealed that recombinant LpxE with mutations in predicted PAP2 catalytic sites (R133A and H197A) were stable and that these residues were in the active site since the mutant proteins failed to dephosphorylate lipid A (56). While this study focused on LpxE, not LpxF, the catalytic sites of these two lipid A phosphatases are well conserved, thus providing further evidence that the PAP2 domain point mutants in our study are likely to have been expressed, but inactive. Additionally, the nature of the mutations within and outside of the PAP2 domain in our study was consistent; basic residues (histidine or arginine) were substituted with alanine and only the mutations within the PAP2 domain phenocopied the *lpxF* null mutant.

The PAP2 superfamily includes phosphatases with diverse substrate specificities, found in both prokaryotes and eukaryotes. These phosphatases are Mg^2+^ independent and share KX_6_RP---PSGH---SRX_5_HX_3_D as their active site motif (57). LpxE and LpxF belong to the LPT (lipid phosphatase/phosphotransferase) branch of transmembrane PAP2 superfamily which is composed of enzymes involved in lipid biosynthesis and structural modification (58). The role of LpxF as a modulator of OMV biogenesis, specifically through the activity of its PAP2 domain, raises questions about whether specific LPT proteins in other taxa of bacteria may regulate OMV biogenesis as well. Additionally, LPT proteins such as LpxE, in *Francisella novicida* and *Aquifex aeolicus*, have been implicated in both lipid A dephosphorylation and dephosphorylation of other lipid substrates, such as undecaprenyl pyrophosphate, linking its activity to peptidoglycan and O-antigen biosynthesis (59). It remains to be seen if LpxF also dephosphorylates other lipid substrates, in addition to lipid A, which may influence membrane destabilization or other aspects of OMV biogenesis in *P. gingivalis* or other species.

While we have demonstrated that lipid A structure plays a significant role in the modulation of OMV production, other studies have identified additional pathways and biomolecules as regulators of OMV biogenesis in *P. gingivalis*. Shortening the O-antigen of LPS through the deletion of the UDP-galactose 4-epimerase gene, *galE*, inhibits OMV production in strain 33277 (60). Deletion of the peptidylarginine deiminase gene, *ppad*, preventing citrullination of bacterial surface proteins, many of which are OMV cargoes, displays a concomitant reduction in OMV production in strain 381 (61). Deletion of the dihydro-sphingosine kinase gene, *DhSphK1,* was also shown to have reduced levels of vesiculation in strain W83, suggesting that membrane lipids other than lipid A also contribute to the OMV biogenesis mechanism in *P. gingivalis* (62). Disruption of the stringent response through the deletion of *rshB* and *rel* genes in strain 381 prevented OMV production, presumably through altered gene regulation, in strain 381 (63). Additional work is needed to determine whether the functions of any of these genes and *lpxF* converge in a single OMV biogenesis mechanism in *P. gingivalis* or whether they operate in parallel. Some common themes do appear to have emerged though, mainly that there is a connection between OMV production and cell envelope composition as well as stress responses.

Along with the previously described contributions of LPS modifications in immune evasion by *P. gingivalis* cells, the results reported here highlight the potential for LPS structural modifications to regulate virulence by modulating OMV production. We demonstrated that OMVs can serve as a vehicle for the delivery of LPS to the extracellular environment where it can bind to TLR4. Furthermore, we showed *P. gingivalis* OMVs are strong activators of TLR2, contributing further to inflammation. It is likely that OMVs released by *P. gingivalis* cells diffuse far from the bacterium where they originated, thus acting as a molecular decoy and promoting inflammation at distant sites (64).

We also showed that reducing *P. gingivalis* OMV production by eliminating lipid A C4′-phosphatase activity results in increased biofilm density, which may have important implications in the context of subgingival plaque, though more work is needed to apply what we have learned to the modulation of cross-species interactions. In conclusion, the findings we report here raise questions about the molecular mechanisms that could link *lpxF* expression or phosphatase activity to environmental cues encountered by *P. gingivalis* in the host, ultimately impacting pathogenesis.

## MATERIALS AND METHODS

### *P. gingivalis* cultures

*P. gingivalis* 33277 and 381 wild-type and mutant strains were obtained from our culture collection (26, 32, 38). All cultures were incubated anaerobically at 37°C. *P. gingivalis* strains were streaked from frozen stock onto TYHK agar, composed of tryptic soy agar (30 g/L), yeast extract (5 g/L), and vitamin K (1 µg/mL), to which hemin was added (10 µg/mL) after autoclaving. Liquid cultures were grown in TYHK broth to which hemin was added fresh before inoculation. For OMV isolations and TEM imaging, starter cultures were sub-cultured twice, with 1:5 dilution each time, and grown 24 h for each culture step. For biofilms, starter cultures were sub-cultured once, with a 1:5 dilution and 24 h incubation. All cultures were incubated anaerobically at 37°C.

### Transmission electron microscopy

Cultures were adsorbed onto Formvar-coated copper mesh grids (Electron Microscopy Sciences) for 2 min, fixed for 1 min in 1% glutaraldehyde, then washed briefly in PBS, followed by sterile water. Grids were stained with 5  mg/mL of phosphotungstic acid for 13 s and air dried. TEM images were acquired using a JEOL JEM-1400 TEM electron microscope at an 80-kV accelerating voltage and an 4K-3K (6.8 µm pixel) sCMOS digital camera and compiled using Adobe Photoshop.

### OMV isolation from liquid cultures

Bacterial strains were grown to stationary phase as described above and growth, recorded by measuring optical density at OD600, showed growth of all the strains to be similar. Culture supernatants from 75 mL of culture, obtained after centrifugation at 7,500 × *g* at 4°C for 10 min, were filtered through a 0.2-µm PES bottle top vacuum filter to remove residual bacteria. OMVs were pelleted from filtered supernatants by ultra-centrifugation at 100,000 × *g* at 4°C for 1 h. Pellets containing OMVs were resuspended in 7 mL of 1× PBS or HEPES buffer (20 mM HEPES, pH 8.0, 150 mM NaCl, 1 M EDTA) and were subjected to another round of ultra-centrifugation at 100,000 × *g* at 4°C for 1 h. Final OMV pellets were resuspended in 1 mL PBS or HEPES buffer and used in subsequent analyses. OMV isolation and quantification were carried out at least twice for each strain at both the UW Seattle and UW Tacoma labs, and data were pooled for reporting in this manuscript.

### Quantitation of OMV protein content

OMV protein concentrations were determined using the Pierce Micro-BCA assay and a Molecular Devices Spectramax ID3 plate reader, following their protocol.  Briefly, each OMV sample or BSA standard was added in triplicate to a 96-well polystyrene plate and absorbance at 562 nm was measured. Each well contained 10 µL OMVs or standard and 200 µL BCA assay working reagent.

### Relative comparison of OMV lipid content

OMVs were also quantified by measuring relative lipid content per volume using a 1,6 diphenyl-1,3,5-hexatriene (DPH; Invitrogen) fluorescence assay (37). For each sample, 25 µL OMV was diluted with 75 µL PBS or Hepes buffer before adding 2 µL of 200 µg/mL DPH. Samples were incubated at room temperature for 30 min prior to measuring DPH fluorescence emission using a Molecular Devices Spectramax ID3 or Biotek Synergy multi-plate reader (excitation 350 nm, emission 452 nm) in a black polysytrene plate. Samples were added to the plate in triplicate.

### Generation of *lpxE* and *lpxF* deletion mutants in Pg W83

We constructed ∆*lpxE::ermF* and ∆*lpxF::ermF* mutants in W83 in a manner similar to their construction in Pg 33277 (26) and Pg 381 (38). Each deletion plasmid construct, containing ~1,000 bp of flanking sequences of the gene of interest on either side of the erythromycin resistance cassette *ermFA*, was introduced into W83 by natural transformation (65). Briefly, 0.5 × 10^9^ W83 was mixed with 1 µg of the deletion plasmid, incubated overnight in a loosely fastened screw-cap Eppendorf tube in an anaerobic chamber, and plated the next morning on TYHK plates containing 5 µg/mL erythromycin. Putative mutant colonies that arose by homologous recombination due to a double cross-over event of the flanking regions on the plasmid into the chromosome were seen 5–6 days later. The deletion mutation in each mutant chosen for analysis was confirmed by PCR and sequencing.

### Generation of *lpxF* point mutant strains in Pg 33277

We constructed individual Pg 33277 point mutations in the *lpxF* gene by site-directed mutagenesis. The amino acid residues targeted were R88, H116, and H157 located within the PAPII domain, as annotated by NCBI, and R132 and H195 outside the PAPII domain, each of which was altered to an alanine residue. To accomplish this, we first constructed a plasmid containing the *lpxF* gene and upstream flanking sequence, from strain 33277, followed by *ermF*, an erythromycin resistance encoding gene, followed by the downstream flanking sequence to generate p-up-flank-*lpxF-ermF*-down-flank (p*lpxF*-Erm). We next mutated the targeted *lpxF* codon in this plasmid using PCR as described below. The point mutations, once generated on p*lpxF-Erm*, were confirmed by sequencing and introduced into the Pg 33277 chromosome by natural trasnformation, as described above. The initial gene-targeting plasmid, p*lpxF-Erm*, was constructed by amplifying fragments encoding (1) *lpxF* 5′ flank and *lpxF* coding sequence using primers SC1 and SC2, listed in Table 1 (2), the *ermF* cassette with SC3-SC4 primers, and (3) the *lpxF* 3′-flanking sequence with primers SC5-SC6. The PCR fragments were digested with appropriate restriction endonucleases (underlined) and ligated into the linearized plasmid pcDNA3.1(-) to yield p*lpxF-Erm*. Next, a two-step PCR amplification procedure was applied to construct each single *lpxF* point mutation. In the first step, PCR fragment pairs were generated using primers (see Table 1) to amplify from p*lpxF-Erm* template as follows:

R88A fragment 1 using primers KB7-KB8, R88A fragment 2 using KB9-KB10;

**TABLE 1:** Primers used in construction of *lpxF* point mutants

SC1	fwd, 5′ flank of lpxF	ACAACTGCTAGCTGAGCATGCGGCGATGGACT
SC2	rev, lpxF ORF	AGACTAGAATTCCTCAGAAGAGCAGGAAGACATAGTACTG
SC3	fwd, ermF	CATTCGAATTCCCGATAGCTTCCGCTATTGC
SC4	rev, ermF	AGATCACTCGAGACGTTTCCGCTCCATCGCCA
SC5	fwd, 3′ flank of lpxF	TCAACACTCGAGCTGAGGAAATTCCTCGGCATCA
SC6	rev, 3′ flank of lpxF	ACAGTAAAGCTTGCGCAAGAGTATGATCTAC
KB7	fwd for fragment 1 of all point mutations	ACAACTGCTAGCTGAGCATGCGGCGATGGACT
KB8	rev, R88A fragment 1	GGGTGGTGCGAAGGGGCGAATCTTGCGAAGAAAGGC
KB9	fwd, R88A fragment 2	GCCTTTCTTCGCAAGATTCGCCCCTTCGCACCACCC (reverse complement of KB8)
KB10	rev for fragment 2 of all point mutations	AGACTAGAATTCCTCAGAAGAGCAGGAAGACATAGTACTG
KB11	rev, H116A fragment 1	GCGATATGTAGTTCGTCGTAGCCCCTGAGATAAATCCG
KB12	fwd, H116A fragment 2	CGGATTTATCTCAGGGGCTACGACGAACTACATATCGC(reverse complement of KB11)
KB13	rev, H157A fragment 1	CCCGGGATAATATCGGTGATGAAAGCCACTCCGATATAAATAC GGC
KB14	fwd, H157A fragment 2	GCCGTATTTATATCGGAGTGGCTTTCATCACCGATATTATCCC GGG (reverse complement of KB13)
KB15	rev, R132A fragment 1	TCCACGTGTAGAATTTATTCGCAAAAATACGGCTCGTA
KB16	fwd, R132A fragment 2	TACGAGCCGTATTTTTGCGAATAAATTCTACACGTGGA (reverse complement of KB15)
KB17	rev, H195A fragment 1	GCATAGACTGCCGACGGAGCGGCAGGGCATGAGGCT
KB18	fwd, H195A fragment 2	AGCCTCATGCCCTGCCGCTCCGTCGGCAGTCTATGC (reverse complement of KB17)

H116A fragment 1 using KB7-KB11, H116A fragment 2 using KB12-KB10;

H157A fragment 1 using KB7-KB13; H157A fragment 2 using KB14-KB10;

R132A fragment 1 using KB7-KB15; R132A fragment 2 using KB16-KB10;

H195A fragment 1 using KB7-KB17; H195A fragment 2 using KB18-KB10.

For the second step, each fragment pair (fragments 1 and 2) was mixed. The two inner primers (KB8 and KB9, for example) were complementary which enabled alignment of the pair, which was then amplified using the common outer primers KB7 and KB10. These PCR fragments were ligated to a plasmid containing the *ermF* cassette and the *lpxF* 3′-flanking sequence to generate the final plasmids containing the *lpxF* 5′-flank, *lpxF* with the point mutation, *ermF* and down-flank. Following the introduction of each of the five plasmids into Pg 33277 by natural transformation (65), putative mutants were selected by plating on TYHK plates containing 5 µg/mL erythromycin. Putative mutant colonies that arose by homologous recombination were seen 5–6 days later. The single-site mutation in each mutant was confirmed by sequencing.

### TLR4 and TLR2 activity assay

HEK293 TLR assays were performed as previously described (26, 32, 41). Briefly, HEK293 cells were plated in 96-well plates and transfected the following day with plasmids encoding human TLRs, NF-κB-dependent firefly luciferase reporter, and β-actin promoter-dependent Renilla luciferase reporter. In the case of human TLR4, 0.002 µg plasmid encoding human TLR4 was co-transfected with 0.0025 µg plasmid encoding human MD-2, a TLR4 co-receptor. In the case of human TLR2, 0.001 µg plasmid encoding human TLR2 was co-transfected with 0.001 µg plasmid encoding human TLR1 and 0.002 µg plasmid encoding human mCD14. At 18– 20 h post-transfection, test wells were stimulated in triplicate for 4 h at 37°C with various doses of sample, which were suspended in Dulbecco’s modified Eagle medium (DMEM) containing 10% human serum. For stimulation with OMVs, a fixed dilution of OMV preparations such as 1:100 in DMEM was used. OMVs were also normalized to lipid content as specified by DPH assay measurements. Luciferase activity was assayed using a dual-luciferase assay reporter system (Promega, Madison, WI). TLR4-dependent NF-κB activation was measured as the ratio of the NF-κB firefly luciferase activity to β-actin promoter-dependent Renilla luciferase activity, which served as an internal standard signifying the number of cells. The data were plotted as the fold difference between the NF-κB activation of the sample and that of the unstimulated control.

### LPS isolation

LPS was isolated from 150 mL of culture using the Tri-reagent protocol as previously described (48). To generate lipid A, dried LPS samples were resuspended in 10 mM sodium acetate (pH 4.5) containing 1% sodium dodecyl sulfate. The solution was heated for 1 h at 100°C, followed by lyophilization overnight. The resulting dried lipid A was resuspended in ice-cold 95% ethanol containing 0.02 N HCl, spun, washed three times in 95% ethanol, and subjected to a final extraction with 1,160 µL of chloroform-methanol-water (1:1:0.9), a biphasic solution that separates residual carbohydrate contaminants into the aqueous phase. The chloroform layer containing lipid A was dried in a fume hood and used for MALDI-TOF MS analyses.

### Analysis of lipid A by MALDI-TOF

MALDI-TOF mass spectrometry was conducted on lipid A samples that were dissolved in 10 µL norharmane matrix, which was prepared by adding 10 mg norharmane to 1 mL of a 1:1 chloroform-methanol solution and mixing well. One microliter of each sample was analyzed in positive ion mode on an AutoFlex II Analyzer (Bruker Daltonics). Data were acquired with a 50 Hz repletion rate, and up to 3,000 shots were accumulated for each spectrum. Instrument calibration and all other tuning parameters were optimized using HP Calmix (Sigma-Aldrich). Data were acquired and processed using Flex Analysis software (Bruker Daltonics).

### Biofilm assay

Cells were obtained from 1.8 mL of subculture by centrifugation at 7,500 × *g* for 5 min. The resulting cell pellet was resuspended in 1 mL of filter-sterilized PBS containing 150 µg carboxyfluorescein succinimidyl ester (CFSE). Cells were incubated with CFSE for 15 min, rotating at room temperature and protected from light. Cells were then centrifuged at 7,500 × *g* for 1 min and resuspended in 1 mL of sterile PBS to wash. Washing was repeated three more times, then the cells were centrifuged as above and resuspended in sterile PBS to obtain a final OD600 of 0.2. Biofilms were formed by adding 750 µL of diluted cell suspension per well to an 18 mm glass coverslip contained in a 12-well polystyrene plate and incubating anaerobically at 37°C for 48 h. After 48 h, the planktonic cells and medium were removed from each well and biofilms were fixed with 4% paraformaldehyde. Each coverslip was mounted with SlowFade Diamond Antifade Mountant (Thermofisher) and sealed with clear nail polish on glass microscope slides.

### Addition of OMVs to biofilms

For *ΔlpxF* biofilms where WT OMVs were added, OMV protein concentration was first determined by BCA assay and then 19 µL of OMVs containing 0.35 mg/mL protein were added per well with bacteria prior to biofilm formation.

### Confocal microscopy

After coverslips were allowed to cure, biofilms were examined and Z-stacks were imaged with a Nikon D-Eclipse C1 Confocal microscope. For visualization of CFSE fluorescence of bacterial cells in biofilm, a green fluorescence channel at 488 nm wavelength. Images were captured with a 100× oil immersion objective lens and 512 × 512 pixel resolution for quantification. A Z projection was generated from the image acquired from confocal imaging. Image analysis was performed with Fiji ImageJ. The pixel intensity was measured for each slice of the z-stack by plotting its Z-Axis profile. Integrated average pixel intensity was determined by the summation of the mean pixel intensities from each slice of the z stack. Integrated pixel intensity for each strain was calculated as a fraction of its associated wild type to quantify the difference of signals across replicates.

### Statistical analysis

Graphpad Prism 10 software was used to compare strain/treatment group results using one- or two-way ANOVA and *post hoc* Tukey tests to identify statistically significant (*P* < 0.05) differences. For DPH and BCA OMV quantification assays, results from three to five independent OMV isolations per strain (*n* = 3–5) were analyzed. For biofilm assays, four to six representative images from six independent biofilms per strain or treatment group (*n* = 6) were analyzed and for HEK TLR4 assays, results from triplicate samples of one representative experiment (*n* = 3) were analyzed.

## SUPPLEMENTAL MATERIAL

Supplemental figures jb.00336-24-s0001.pdf Fig. S1 to S4.

ASM does not own the copyrights to Supplemental Material that may be linked to, or accessed through, an article. The authors have granted ASM a non-exclusive, world-wide license to publish the Supplemental Material files. Please contact the corresponding author directly for reuse.
