# Supplementary material for: Lipid a remodeling modulates outer membrane vesicle biogenesis by Porphyromonas gingivalis
Source: J Bacteriol. 2024 Dec 11;207(1):e00336-24. doi: 10.1128/jb.00336-24 (PMC11784228; doi:10.1128/jb.00336-24)
Supplement: Supplemental figures — Fig. S1 to S4. [file jb.00336-24-s0001.pdf]

Figure S1.

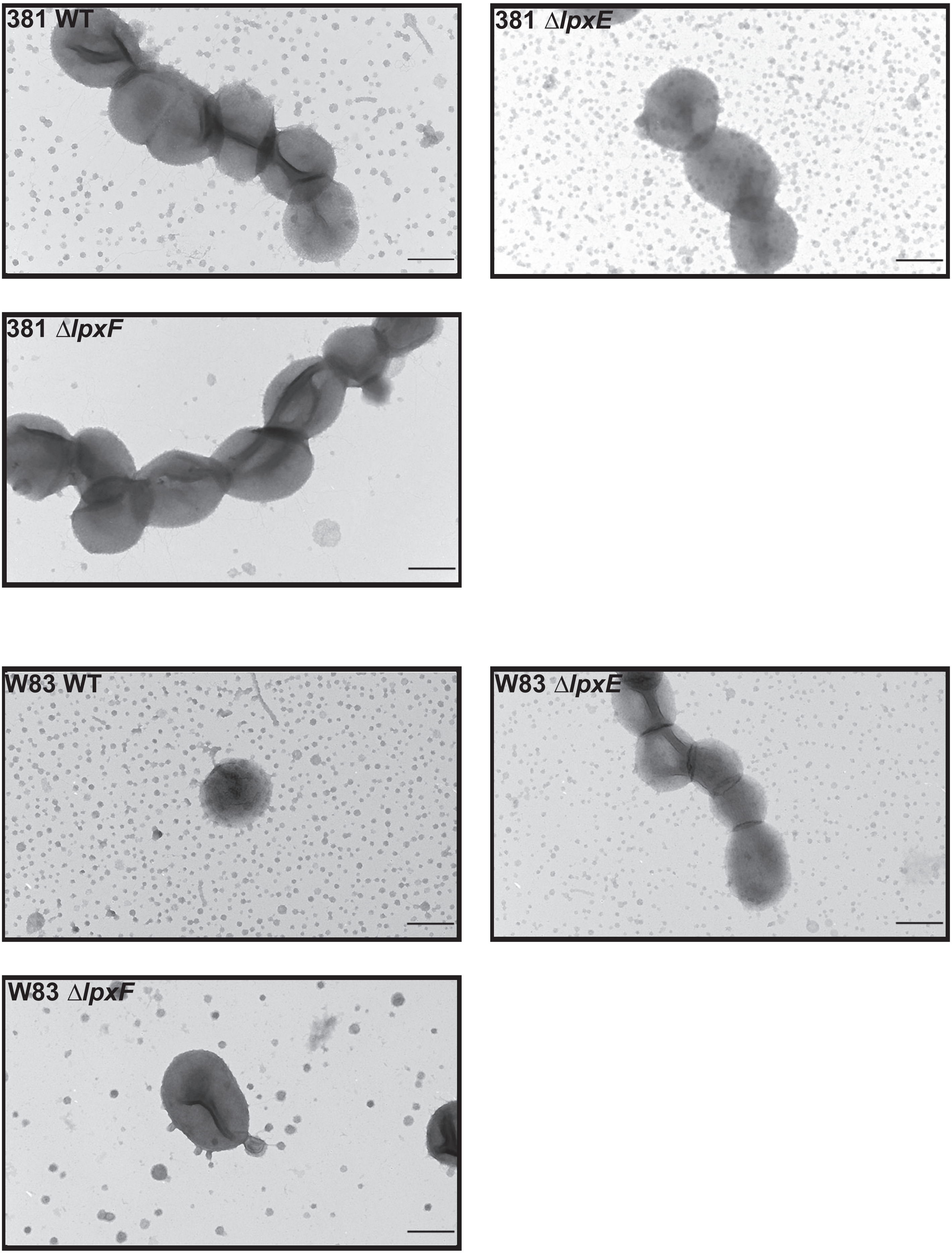

Figure S1. Deletion of *lpxF* in *P. gingivalis* 381 and W83 strains impairs OMV biogenesis. TEM images of whole broth cultures used to compare abundances of OMVs produced by WT 381 or WT W83 and their lipid A phosphatase mutants is shown. Scale bars represent 500 nm.

Figure S2. **MALDI-TOF MS of *lpxF* site-directed mutants**

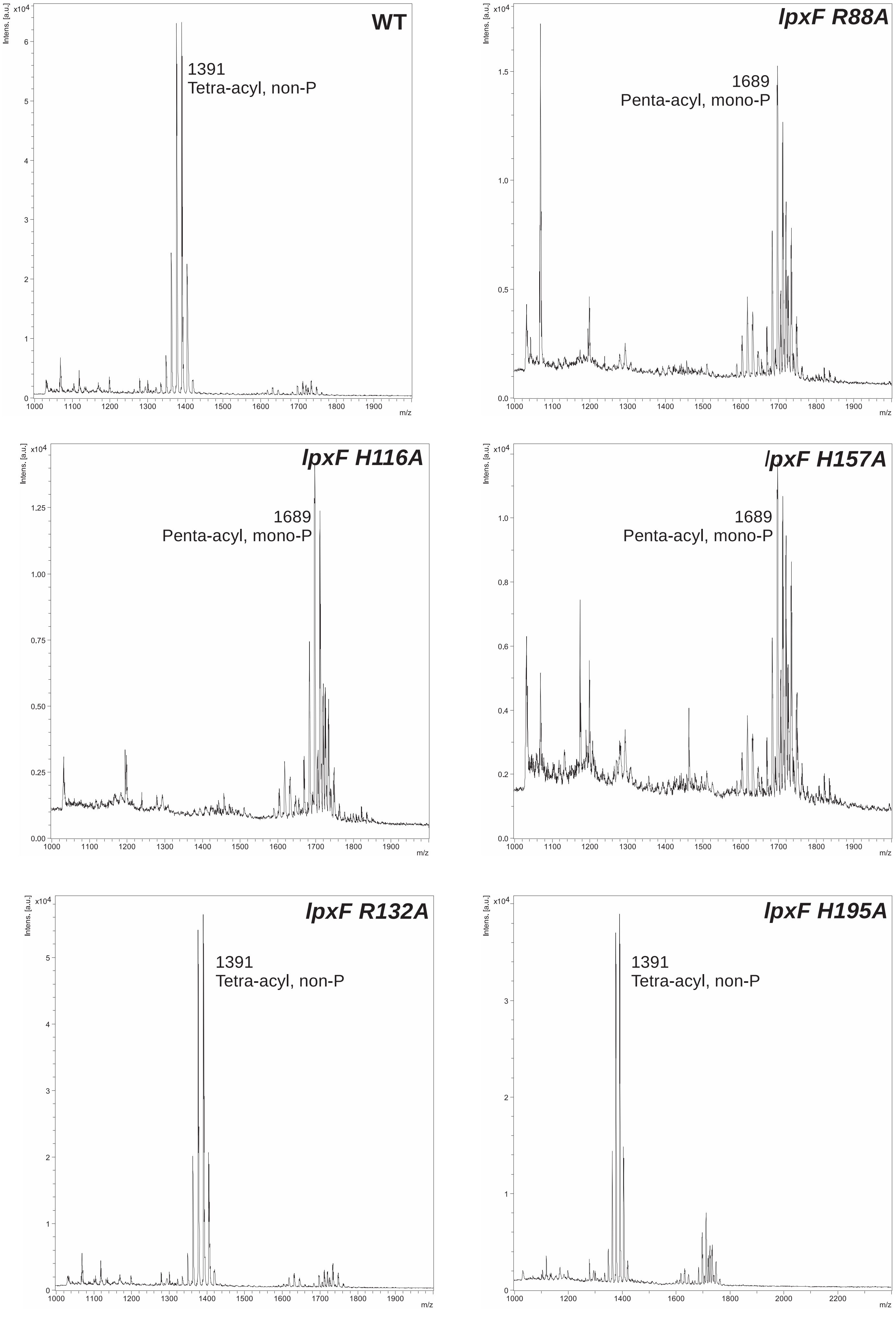

Figure S2. Lipid A in strains with point mutations within the PAPII domain of LpxF is penta-acylated mono-phosphorylated. Lipid A of strains harboring lpxF with point mutations in R88A, H116A and H157A is predominantly penta-acylated mono-phosphorylated (m/z 1689), as detected by MALDI-TOF MS, unlike WT and strains that have mutations outside the PAPII domain, namely R132A and H195A, which possess lipid A that is largely tetra-acylated non-phosphorylated (m/z 1391). MALDI-TOF mass spectrometry of purified lipid A was conducted in positive-ion mode. Numbers refer to the m/z ratio of the predominant peak in each lipid A cluster.

Figure S3.

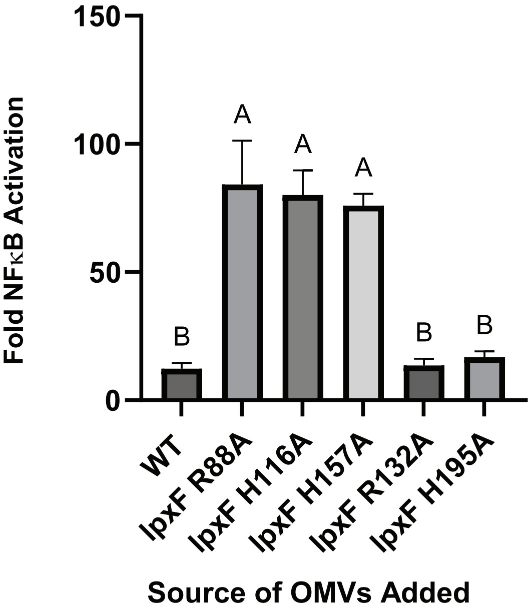

Figure S3. OMVs from strains with point mutations within the PAPII domain of LpxF activate TLR4 at a significantly higher level than OMVs from WT or strains harboring point mutations outside of the PAPII domain. OMVs were normalized to lipid content of wild-type OMVs, as measured by DPH assay, and subjected to a HEK293 TLR4 luciferase assay. Fold NF-kB stimulation of OMV treated cells relative to the unstimulated control is plotted on the y-axis. The results are means +/- SD of triplicate samples from one of two independent experiments, and different letters above bars indicate statistically significant differences between treatments ($p<0.05$) determined by one-way ANOVA and post hoc Tukey test.

Figure S4.

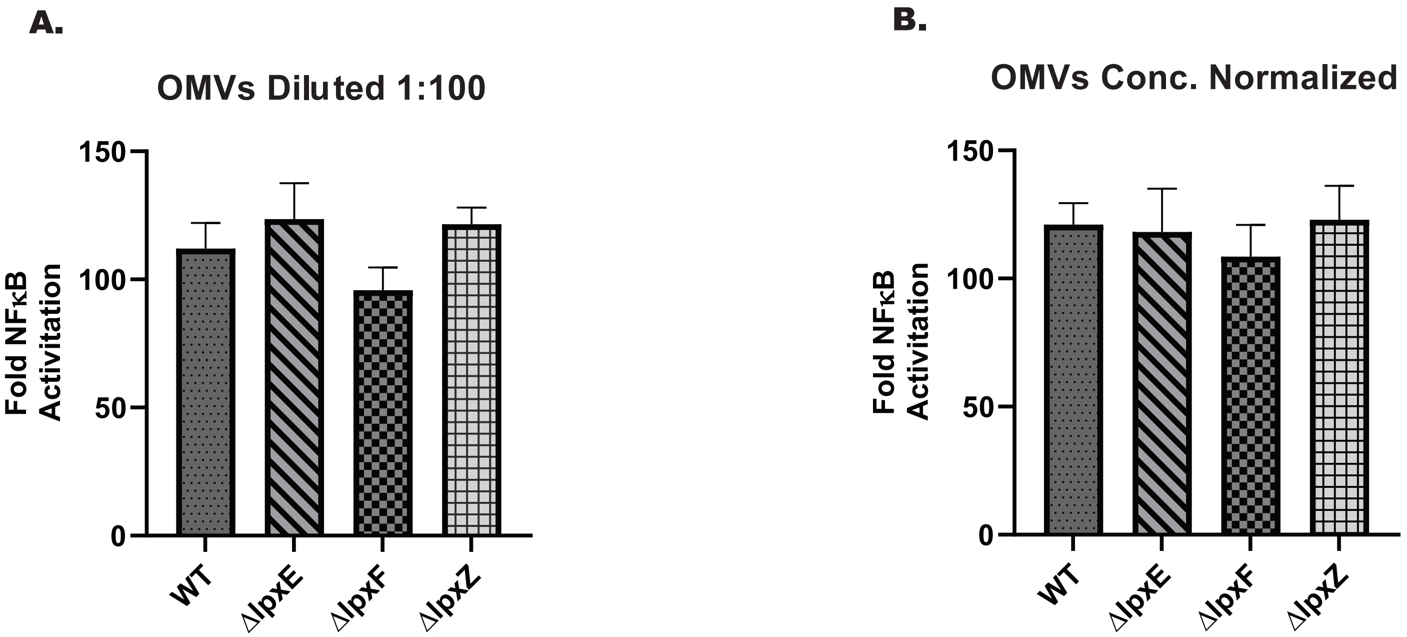

Figure S4. *P. gingivalis* OMVs stimulate TLR2/TLR1. HEK293 cells expressing TLR2,TLR1, and CD14 were exposed to OMVs that were either diluted 1:100 after isolation or normalized by quantity to lipid content of WT OMVs as measured by DPH assay. A luciferase reporter assay was used to determine fold NF-κB stimulation of OMV exposed cells relative to the unstimulated control. The results are means +/- SD of triplicate samples from two independent experiments. No significant difference between treatments was found by one-way ANOVA and post hoc Tukey test.
